# Physicians’ Perceptions of and Barriers to Cardiopulmonary Rehabilitation for Heart Failure Patients in Saudi Arabia: A Cross-Sectional Study

**DOI:** 10.3390/ijerph192215208

**Published:** 2022-11-17

**Authors:** Abdulelah M. Aldhahir, Munyra Alhotye, Jaber S. Alqahtani, Saeed M. Alghamdi, Abdullah S. Alsulayyim, Abdullah A. Alqarni, Eidan M. Alzahrani, Rayan A. Siraj, Hassan Alwafi

**Affiliations:** 1 Respiratory Therapy Department, Faculty of Applied Medical Sciences, Jazan University, Jazan 82511, Saudi Arabia; 2 Department of Respiratory Therapy, College of Applied Medical Sciences, King Saud Bin Abdulaziz University for Health Sciences, Riyadh 12211, Saudi Arabia; 3 Department of Respiratory Care, Prince Sultan Military College of Health Sciences, Dammam 32210, Saudi Arabia; 4 Respiratory Care Program, Clinical Technology Department, Faculty of Applied Medical Sciences, Umm Al-Qura University, Makkah 24211, Saudi Arabia; 5 National Heart and Lung Institute, Imperial College London, London SW7 2BX, UK; 6 Department of Respiratory Therapy, Faculty of Medical Rehabilitation Sciences, King Abdulaziz University, Jeddah 22230, Saudi Arabia; 7 Department of Physiotherapy, Prince Sultan Military College of Health Sciences, Dammam 32210, Saudi Arabia; 8 Department of Respiratory Therapy, College of Applied Medical Sciences, King Faisal University, Al-Hasa 36291, Saudi Arabia; 9 Faculty of Medicine, Umm Al-Qura University, Mecca 36291, Saudi Arabia

**Keywords:** cardiopulmonary rehabilitation, heart failure, Saudi Arabia, physicians

## Abstract

Background: Cardiopulmonary rehabilitation (CR) serves as a core component of the management strategy for patients with heart failure (HF). CR is administered by multidisciplinary healthcare providers, but their perceptions toward delivering CR to HF patients, and the factors and barriers that might influence referral, have not been studied. This study aims to assess physicians’ perceptions toward delivering CR programs to HF patients and identify factors and barriers that might influence their referral decisions. Methods: Between 15 February and 5 June 2022, a cross-sectional online survey with ten multiple-choice items was distributed to all general and cardiac physicians in Saudi Arabia. The characteristics of the respondents were described using descriptive statistics. Percentages and frequencies were used to report categorical variables. The statistical significance of the difference between categorical variables was determined using the chi-square (2) test. Logistic regression was used to identify referral factors. Results: Overall, 513 physicians (general physicians (78%) and cardiac doctors (22%)) completed the online survey, of which 65.0% (*n* = 332) were male. Of the general physicians, 236 (59%) had referred patients with HF to CR. Sixty-six (58%) of the cardiac doctors had referred patients with HF to CR. A hospital-supervised program was the preferred mode of delivering CR programs among 315 (79%) general physicians, while 84 (74%) cardiac doctors preferred to deliver CR programs at home. Apart from the exercise component, information about HF disease was perceived by 321 (80%) general physicians as the essential component of a CR program, while symptom management was perceived by 108 (95%) cardiac doctors as the essential component of a CR program. The most common patient-related factor that strongly influenced referral decisions was “fatigue related to disease” (63.40%). The availability of CR centers (48%) was the most common barrier preventing the referral of patients to CR. Conclusions: CR is an effective management strategy for HF patients, but the lack of CR centers is a major barrier to the referral of patients. A hospital-supervised program is the preferred method of delivering CR from the general physicians’ perspective, while cardiac doctors prefer home-based CR programs. Apart from the exercise component, information about HF disease and symptom management is essential components of CR programs from general physicians’ and cardiac doctors’ perspectives, respectively.

## 1. Introduction 

Heart failure (HF) is a clinical condition in which the heart fails to pump enough blood to meet the body’s demands for blood, eventually causing a reduction in organ perfusion and some other serious complications [1]. HF has been the leading cause of death and disability in the last 20 years and is a highly prevalent disorder worldwide, with its high morbidity and mortality rate leading to increased healthcare costs [2]. Although daily symptoms of HF, in most cases, are nonspecific and may coexist with those of other clinical conditions, the worsening of common symptoms (e.g., activity limitation and shortness of breath) in patients with HF is associated with reduced exercise capacity, leading to frequent hospital admissions [1,3,4].

The goals of treatment in patients with HF are to reduce exacerbations, hospitalization, and hospital length of stay by improving functional capacity and quality of life and reducing mortality [1]. In addition to pharmacologic treatments, the use of non-pharmacologic interventions (e.g., sodium restriction, nutritional support, and exercise training) is recommended to prevent the symptoms of HF [1]. CR serves as a core component of non-pharmacologic management strategies for patients with HF [1]. CR for patients with HF, also called cardiac rehabilitation, is a comprehensive multidisciplinary program that aims to improve functional capacity, exercise duration, and quality of life in patients with HF [1]. Based on the patient’s needs and the resources available, the CR program for patients with HF should comprise a physical assessment, patient education, nutritional and psychological support, and physical exercise training. Given the variety of services that should be included in the CR program for patients with HF, CR programs should be run by a multidisciplinary team. This team should include physicians (preferably cardiologists), physiotherapists, nurses, clinical pharmacists, dieticians, social workers, and additional subspecialty providers [2].

Exercise training and other components of CR are effective and safe in patients with HF [5]. Studies have demonstrated that the application of CR for patients with HF is associated with a reduction in mortality and hospitalizations and improved functional capacity, exercise duration, and health-related quality of life [6,7,8,9]. Furthermore, it has been demonstrated that CR for patients with HF can improve peak oxygen consumption [10,11,12,13,14,15]. Although these findings strongly suggest that the use of CR is recommended for patients with HF to reduce hospitalization and improve quality of life, CR services are still underutilized globally [5].

In Saudi Arabia, specific CR programs for patients with HF are not well utilized, likely due to limited capacity, a lack of staff training, a lack of CR centers, and the inability of CR centers to maintain patient safety standards [16,17]. In addition, the perceptions of healthcare professionals, particularly physicians, about delivering CR programs among HF patients in Saudi Arabia have not been studied before. Therefore, we aim to determine physicians’ perceptions toward delivering CR programs to patients with HF and to identify barriers and factors that might influence the decisions of physicians to refer HF patients to CR programs in Saudi Arabia.

## 2. Methods

### 2.1. Study Design

Between 15 February 2022 and 5 June 2022, a cross-sectional survey was conducted and distributed using an online platform (Survey Monkey).

### 2.2. Questionnaire Tool

We used a modified version of a survey that was previously designed, created, and validated by Aldhahir et al. [18,19,20,21,22,23,24]. The survey consisted of ten closed multiple-choice questions with free text spaces for extra comments.

The purpose of the study, as well as information on the lead investigator, was supplied to participants before they began answering the questionnaire. Furthermore, no personal information was collected; participants’ consent was obtained by asking if they were happy to complete the online survey, and if they were, the survey was completed. “By answering yes in completing the survey question, you freely agree to engage in this study and offer your agreement to utilize your anonymous data for research purposes”, the survey stated. The survey took between three and five minutes to complete. The questionnaire consisted of two pages of structured responses divided into three sections, each with multiple-choice answers. The first section contained the respondents’ demographic information, including gender, profession, geographic location, years of experience, responsibilities in the management of patients with HF, and whether physicians had ever referred HF patients to cardiopulmonary rehabilitation programs before. The second section consisted of three questions asking about physicians’ perceptions of cardiopulmonary rehabilitation. The first question had five statements regarding the effectiveness of cardiopulmonary rehabilitation with HF patients and used a 5-point Likert scale ranging from 1 “strongly disagree” to 5 “strongly agree”. The second question asked about additional components of cardiopulmonary rehabilitation aside from the exercise component, and the third question was about the ideal method of delivering cardiopulmonary rehabilitation to HF patients. The third section included two questions regarding patient-related factors that influence referral decisions and process-related factors that influence the decision not to refer patients with HF. These questions used influence as a grading tool (no influence, some influence, and strong influence).

### 2.3. Study Population and Sampling Strategy

The study participants were recruited using a convenience sampling technique. The main target populations were general physicians and cardiac doctors who worked with HF patients or had prospective contact with this population. To reach a larger number of physicians working in Saudi Arabia, the survey was distributed through professional committees handling cardiac disorders, such as the Saudi Heart Association and Saudi Cardiac Interventional Society, as well as social media (Twitter, WhatsApp, and Telegram). The study inclusion criteria were clearly stated in the invitation letter of the study.

### 2.4. Sample Size

Sample size calculation was not required, as this was an exploratory study design. 

### 2.5. Ethical Approval

Institutional Review Board approval for the study was obtained from Jazan University, reference number (REC-43/03/041).

### 2.6. Statistical Analysis

Data were collected and analyzed using the Statistical Package for Social Sciences (SPSS software, Version 25, IBM, Armonk, NY, USA). The categorical variables were reported and presented in percentages and frequencies. A Chi-square (χ^2^) test was used to assess the statistically significant difference between categorical variables. Logistic regression was used to predict whether years of experience would be a referring factor. Statistical significance was considered if *p* < 0.05.

## 3. Results

Overall, 513 physicians, comprising 332 males (65%) and 181 females (35%), completed the online survey between 15 February 2022 and 5 June 2022. Out of these, general physicians accounted for 78% and cardiac doctors accounted for 22% (Table 1). The survey attempted to reach all regions of the Kingdom of Saudi Arabia. Respondents were distributed across the Kingdom’s regions as follows: 138 (27%) were from the central region; 134 (26%) were from the western region; 113 (22%) were from the eastern region; 82 (16%) were from the southern region, and 46 (9%) were from the northern region (Table 1). The majority of general physicians had more than ten (32%) or five to six (16%) years of clinical experience in caring for patients with HF, while the majority of cardiac doctors had more than ten (40%) or five to six (20%) years of clinical experience in caring for patients with HF (Table 1). The most common responsibilities of general physicians in caring for HF patients were diagnosis (79%), followed by urgent assessment (61%) and medication checks (60%) (Table 1). The most common responsibilities of cardiac doctors in caring for HF patients were diagnosis (95%), followed by in-patient treatment (85%) and ongoing management (75%).

### 3.1. Cardiopulmonary Rehabilitation Referral Rate by Physicians’ Specialty

Within the general physician group, 236 (59%) had referred patients with HF to a cardiopulmonary rehabilitation program, and 99 (25%) or 64 (16%) had not referred or were not sure if they ever had referred a patient with HF to a cardiopulmonary rehabilitation program, respectively (Table 2). General physicians with more years of clinical experience with HF patients were one time more likely to refer patients with HF to a cardiopulmonary rehabilitation program (OR: 1.3 (95% CI 1.2–1.5): *p* = 0.002)

Within the cardiac doctor group, 66 (58%) had referred patients with HF to a cardiopulmonary rehabilitation program, and 33 (29%) or 15 (13%) had not referred or were not sure if they ever had referred a patient with HF to a cardiopulmonary rehabilitation program, respectively (Table 2). Cardiac doctors with more years of clinical experience with HF patients were two times more likely to refer patients with HF to a cardiopulmonary rehabilitation program (OR: 2 (95% CI 1.4–2.8): *p* < 0.001).

Overall, there was no statistically significant difference in the referral rate between the general physician and cardiac doctor groups (*p* > 0.05). 

### 3.2. Opinions on Referring Patients with HF, Mode of Delivery, and Components of Cardiopulmonary Rehabilitation

Out of 399 general physicians, 194 (49%) strongly agreed and 134 (34%) agreed that CR would improve patients’ physical fitness. Additionally, 152 (38%) strongly agreed and 143 (36%) agreed that CR would reduce breathlessness in patients with HF. Most general physicians strongly agreed (147–37%) or agreed (127–32%) that CR would improve HF patients’ palpitation and fatigue. Out of 399 general physicians, 184 (46%) strongly agreed and 124 (31%) agreed that CR would improve HF patients’ ability to perform daily activities, and 75 (19%) strongly agreed and 118 (30%) agreed that CR would reduce hospital readmission (Table 3).

Out of 114 cardiac doctors, 52 (45%) strongly agreed and 57 (50%) agreed that CR would improve patients’ physical fitness. Additionally, 33 (29%) strongly agreed and 68 (59%) agreed that CR would reduce breathlessness in patients with HF. Most cardiac doctors strongly agreed (20–17%) or agreed (65–57%) that CR would improve HF patients’ palpitation and fatigue. Out of 114 cardiac doctors, 54 (48%) strongly agreed and 55 (48%) agreed that CR would improve HF patients’ ability to perform daily activities, and 9 (8%) strongly agreed and 46 (41%) agreed that CR would reduce hospital readmission (Table 3).

Out of 399 general physicians, 315 (79%) believed that the preferred method to deliver a CR program is a hospital-supervised program, followed by 223 (56%) who preferred at-home programs. In contrast, a tailored program with healthcare provider support through the phone was the least preferred method to deliver CR programs, by 152 participants (38%) (Table 3). 

Out of 114 cardiac doctors, 84 (74%) believed that the preferred means to deliver a CR program is at home, followed by 82 (72%) who preferred hospital-supervised programs. In contrast, an online program with healthcare provider support was the least preferred method to deliver CR programs, chosen by 28 participants (25%). 

Information about HF disease, followed by information about medications and smoking cessation, were the essential components of CR programs from general physicians’ perspective, aside from the exercise component, being chosen by 321 (80%), 280 (70%), and 270 (68%) participants, respectively (Table 3).

Symptom management, followed by stress management and information about medications, were the essential components of CR programs from cardiac doctors’ perspective, aside from the exercise component, being chosen by 108 (95%), 96 (84%), and 95 (83%), participants, respectively (Table 3).

### 3.3. Patient-Related Factors That Influence Referral Decision to Cardiopulmonary Rehabilitation

**Figure 1:**
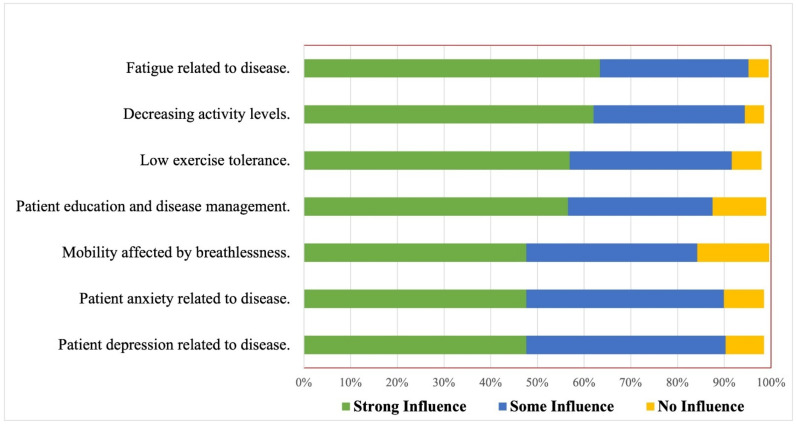
Patient-related factors that influence referral decision to cardiopulmonary rehabilitation, using strong, some or no influence as a grading tool (*n* = 513).

The patient-related factor that strongly influenced all respondents’ referral decisions for CR included fatigue related to disease (63.40%), followed by decreased activity levels (62%) and low exercise tolerance (57%) (Figure 1). For each group (general physicians and cardiac doctors), patient-related factors that strongly influenced referral decisions for CR are reported in Supplementary Figure S1.

### 3.4. Cardiopulmonary Rehabilitation Referral Barriers

**Figure 2:**
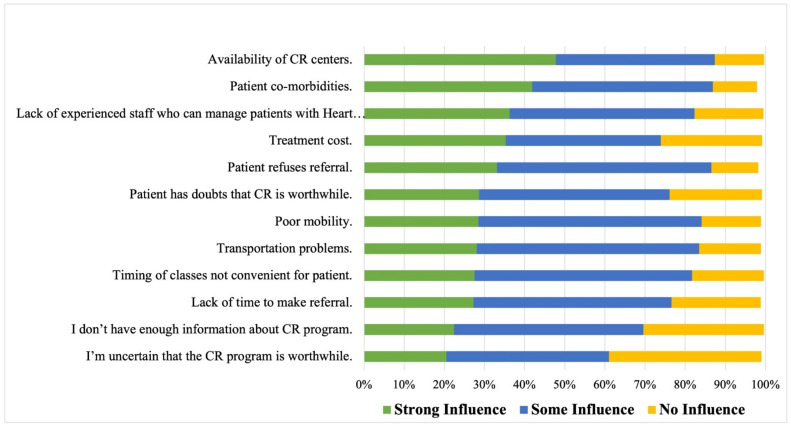
Barriers of not referring patients with Heart Failure to cardiopulmonary rehabilitation, using influence graded as no, some or strong influence (*n* = 513).

The process-related factors that strongly influenced physicians’ decisions not to refer patients with HF to CR included the availability of CR centers (48%), followed by patient comorbidities (42%) and a lack of experienced staff who could manage patients with HF (36%) (Figure 2). For each group (general physicians and cardiac doctors), process-related factors that strongly influenced physicians’ decisions not to refer patients with HF to CR are reported in Supplementary Figure S1.

## 4. Discussion

To the best of our knowledge, this is the first national survey that assesses the insights of general and cardiac physicians into the benefits of CR programs and their experience with the referral process, as well as their referral considerations regarding CR programs for patients living with HF in Saudi Arabia. Physicians of both specialties perceived CR as an effective management strategy for improving patients’ physical activity and disease-related symptoms, and for reducing hospital readmission among patients with HF. In addition, physicians with more clinical experience in caring for HF patients were more likely to refer patients to CR programs. Moreover, general physicians preferred the hospital-supervised CR program for this population, with information about HF and medications as essential components of the program, aside from exercise training. On the other hand, cardiac doctors perceived home-based CR as the preferred method of delivering CR for patients with HF, with symptoms and stress management being essential components of the CR program, aside from exercise training.

Cardiac rehabilitation is considered an effective and safe non-pharmacological intervention for patients with HF [25,26]. It helps to mitigate HF disease-related symptoms such as shortness of breath and fatigue; it improves exercise capacity and cardiopulmonary fitness and quality of life, and it reduces HF-related hospitalization [27,28]. In our study, physicians perceived CR as an effective management strategy for HF patients, and this has been demonstrated through a high referral rate to the program. Furthermore, having several years of clinical experience in dealing with HF patients was a crucial factor in influencing the decision to refer patients with HF to CR. These findings are in line with a previous study that highlighted that the level of experience as a healthcare professional might be a factor in enhancing knowledge about cardiac rehabilitation programs [29].

In addition, our study reported that more than half of the physicians of both specialties had referred HF patients to CR programs, with cardiologists making more referrals compared to general physicians. We found that cardiologists are almost two times more likely to refer patients with HF to CR compared to general physicians. This is in line with previous research conducted by Barber et al., who reported that the referral rate to CR varies by healthcare provider specialty. Barber et al. found that patients who were managed by a cardiac specialist were more likely to be referred to a CR program [30]. Moreover, another study reported that general physicians are less aware of the referral process [31]. Reasons behind this might be the fact that physicians from other specialties lack familiarity with the referral guidelines or the availability of CR centers, despite their existence within the healthcare facility. Moreover, cardiologists may be more likely to have more clinical training and experience with CR and are routinely more involved in CR programs compared to other specialties [31], hence contributing to an increased referral rate. Furthermore, physicians with more clinical experience were more likely to refer patients to CR programs, which may indicate adequate knowledge of CR and its benefits, greater familiarity with resources and guidelines, and higher exposure to HF cases. Therefore, it is crucial to provide more education related to HF management, and disseminating current clinical guidelines to other physicians from different specialties will increase their referral rates.

One of the main barriers preventing the referral of HF patients to CR is the lack of availability of CR centers. Few CR centers provide this service in Saudi Arabia, and they only accommodate a limited number of patients with cardiovascular disease [16]. Moreover, our results also agree with a previous study that explored the barriers to establishing pulmonary rehabilitation programs in Saudi Arabia, which highlights the lack of capacity in healthcare facilities as one of the main barriers [32]. This reveals a considerable shortcoming in current practice and highlights the need to formulate and establish comprehensive CR programs that meet international guidelines across the country. However, it is worth noting that CR could be integrated within current clinical sites with multidisciplinary cooperation [29].

Another major barrier to the referral process in this study is patient comorbidities. Patients with HF tend to have coexisting chronic conditions, including pulmonary dysfunction, hypertension, diabetes mellitus, musculoskeletal conditions, cognitive limitations, and depression [33]. Furthermore, patients with this condition generally have age-related limitations, which include frailty, physical disability, and hearing and visual impairments [34]. In addition, symptoms related to HF, such as fatigue and shortness of breath, may affect patients’ capacity to exercise and could limit their referral to the program. These findings are in line with previous studies that investigated the barriers to CR in patients with HF, which reported that HF patients with comorbidities are less likely to enroll in CR programs [35,36,37]. Therefore, there is a need to deliver a tailored CR program according to the patient’s capacity. Moreover, the mode and intensity of exercises should be adjusted according to the patient’s personal fitness level, preferences, goals, and medical condition to improve participation in the program.

In our study, most general physicians reported that a hospital-based, supervised program is the most suitable way to deliver CR for these patients. Interestingly, the majority of cardiac physicians claimed that a home-based program was the most suitable way to deliver the program. This might be attributed to the limited number of CR centers in the country [16], and this mode is not always accessible or convenient for many patients, especially those who live in rural areas or those who do not wish to participate in group programs [38,39]. It is also possible that the COVID-19 pandemic increased the preference for home-based CR programs, as most face-to-face services were forced to close temporarily to keep patients safe. In this situation, home-based CR seems to be an alternative option to deliver the program, given the limited number of experienced staff and available CR centers. It is important, however, to highlight that home-based CR is effective as a conventional CR program in improving disease-related symptoms such as dyspnea, fatigue, exercise capacity, and overall quality of life in this population [40,41]. Furthermore, a home-based program could offer greater accessibility and can improve program uptake.

In this study, physicians of both specialties reported that patient education about HF, symptom management, and medication use should be implemented through the CR program, in addition to the exercise component. This is in line with the current BACR and the American College of Cardiology (ACC), American Heart Association (AHA), and Heart Failure Society of America (HFSA) official guidelines on the main components of CR [42,43]. Patients suffering from HF had limited levels of knowledge and lacked understanding of their condition and how to manage their symptoms, which could be a factor in their frequent hospital readmissions [44,45]. Furthermore, HF could lead to increased mortality and a burden on clinical services. In addition, it has been reported that patients with HF had a limited understanding of the purpose of their medication and how to treat symptoms, and they could not tell the difference between disease symptoms and the side effects of their medication [46]. Therefore, patient education is an essential component as it helps them to manage their symptoms and maintain control over disease-related episodes, which improves their overall health and well-being.

### Limitations

Some limitations of this study should be highlighted. Firstly, this study was based on a convenience sampling strategy, which has the potential for selection bias. Moreover, we only recruited general and cardiac physicians in this study, which could indicate a possibility of bias. However, this was an exploratory study designed to obtain initial opinions and thoughts regarding CR from those who provide direct care for this population. Another limitation is that the number of cardiac physicians involved in this study was considered low, and this was due to the low number of cardiologists in the Kingdom. Moreover, the study would have benefited from more in-depth structured interviews to gather more details regarding the barriers to CR programs. Lastly, this study was conducted during the COVID-19 pandemic, which might be a factor that influenced the decision to choose an alternative mode of program delivery.

## 5. Conclusions 

CR’s benefits are well recognized among physicians, irrespective of their specialties, which has been demonstrated through the high referral rate to CR. The lack of CR centers and patients’ comorbidities were the main barriers to the referral of HF patients to CR programs. The hospital-supervised program was the most suitable means of delivering CR, while smoking cessation was the most essential component of CR programs, apart from the exercise component.

## Figures and Tables

**Table 1:** Demographic data and characteristics of all study respondents (*n* = 513).

Demographic Variables	General Physicians	Cardiac Doctors
*n* (%)	399 (78%)	114 (22%)
Gender		
Male	249 (62%)	83 (73%)
Female	150 (38%)	31 (27%)
Geographic Location		
Eastern region	87 (22%)	26 (23%)
Central region	108 (27%)	30 (26%)
Western region	105 (26%)	29 (25%)
Southern region	64 (16%)	18 (16%)
Northern region	35 (9%)	11 (10%)
Year of experience with HF patients		
<1 year	21 (5%)	1 (1%)
1–2 years	41 (10%)	4 (4%)
3–4 years	56 (14%)	4 (4%)
5–6 years	64 (16%)	23 (20%)
7–8 years	46 (12%)	22 (19%)
9–10 years	43 (11%)	14 (12%)
>10 years	128 (32%)	46 (40%)
Responsibilities of care for HF patients		
Diagnosis	314 (79%)	108 (95%)
Urgent assessments	243 (61%)	83 (73%)
Non-urgent care	196 (49%)	69 (61%)
Ongoing management	212 (53%)	86 (75%)
Admission prevention	165 (41%)	57 (50%)
Medication checks	240 (60%)	32 (28%)
Prescribing	211 (53%)	70 (61%)
Oxygen therapy	144 (37%)	30 (26%)
In-patient treatment	215 (54%)	97 (85%)
Outpatient clinics	110 (28%)	67 (59%)
Primary care	148 (37%)	26 (23%)
Others	5 (1%)	12 (11%)

Data are presented as frequencies and percentages.

**Table 2:** Patients’ referral rate by general physician and cardiac doctor groups (*n* = 513).

Item	Frequency (%)
Patients’ Referral	
General physician	
Yes	236 (59%)
No	99 (25%)
Not sure	64 (16%)
Cardiac doctor	
Yes	66 (58%)
No	33 (29%)
Not sure	15 (13%)

Data are presented as frequencies and percentages.

**Table 3:** Perceptions of referring patients with HF to cardiopulmonary rehabilitation, mode of delivery, and components of cardiopulmonary rehabilitation (*n* = 513).

Item	General Physicians	Cardiac Doctors
Perception of referring HF patients to CR		
I believe CR will improve patients’ physical fitness		
Strongly agree	194 (49%)	52 (45%)
Agree	134 (34%)	57 (50%)
Neutral	49 (12%)	1 (1%)
Disagree	3 (0.7%)	1 (1%)
Strongly disagree	19 (5%)	3 (3%)
I believe CR will reduce patients’ breathlessness		
Strongly agree	152 (38%)	33 (29%)
Agree	143 (36%)	68 (59%)
Neutral	70 (18%)	8 (7%)
Disagree	14 (4%)	4 (4%)
Strongly disagree	20 (5%)	1 (1%)
I believe CR will improve patients’ palpitation & fatigue		
Strongly agree	147 (37%)	20 (17%)
Agree	127 (32%)	65 (57%)
Neutral	98 (25%)	27 (24%)
Disagree	12 (3%)	1 (1%)
Strongly disagree	15 (4%)	1 (1%)
I believe CR will improve patients’ ability to perform daily actives		
Strongly agree	184 (46%)	54 (48%)
Agree	124 (31%)	55 (48%)
Neutral	65 (16%)	2 (2%)
Disagree	6 (2%)	1 (1)
Strongly disagree	20 (5%)	2 (2%)
I believe CR will reduce hospital readmission		
Strongly agree	75 (19%)	9 (8%)
Agree	118 (30%)	46 (41%)
Neutral	96 (24%)	37 (32%)
Disagree	40 (10%)	6 (5%)
Strongly disagree	70 (18%)	16 (14%)
The best way to deliver CR program for HF patients		
At home	223 (56%)	84 (74%)
In hospital-supervised program	315 (79%)	82 (72%)
Online program with healthcare provider support	189 (47%)	28 (25%)
Tailored program with healthcare provider support through phone	152 (38%)	53 (46%)
Components of CR program aside from exercise component		
Information about HF disease	321(80%)	93 (82%)
Weight management	269 (67%)	93 (82%)
Stress management	265 (66%)	96 (84%)
Information about medications	280 (70%)	95 (83%)
Symptoms management	261 (65%)	108 (95%)
Smoking cessation	270 (68%)	91 (80%)
Others	7 (2%)	23 (20%)

Data are presented as frequencies and percentages. Abbreviations: HF, heart failure; CPF, cardiopulmonary rehabilitation.

## Data Availability

Data are available upon reasonable request.

## Supplementary Materials

The following supporting information can be downloaded at: https://www.mdpi.com/article/10.3390/ijerph192215208/s1, Figure S1: Patient-related factors that influence referral decision to cardiopulmonary rehabilitation from cardiac doctors’ perspective, using strong, some or no influence as a grading tool (*n* = 114). Figure S2. Barriers of not referring patients with Heart Failure to cardiopulmonary rehabilitation from cardiac doctors’ perspective, using influence graded as no, some or strong influence. (*n* = 114). Figure S3. Patient-related factors that influence referral decision to cardiopulmonary rehabilitation from general physicians’ perspective, using strong, some or no influence as a grading tool (*n* = 399). Figure S4: Barriers of not referring patients with Heart Failure to cardiopulmonary rehabilitation from general physicians’ perspective, using influence graded as no, some or strong influence. (*n* = 399).
