# Supplementary material for: Physicians’ Perceptions of and Barriers to Cardiopulmonary Rehabilitation for Heart Failure Patients in Saudi Arabia: A Cross-Sectional Study
Source: Int J Environ Res Public Health. 2022 Nov 17;19(22):15208. doi: 10.3390/ijerph192215208 (PMC9690397; doi:10.3390/ijerph192215208)

Figure S1. Patient-related factors that influence referral decision to cardiopulmonary rehabilitation from cardiac doctors’ perspective, using strong, some or no influence as a grading tool (n=114).

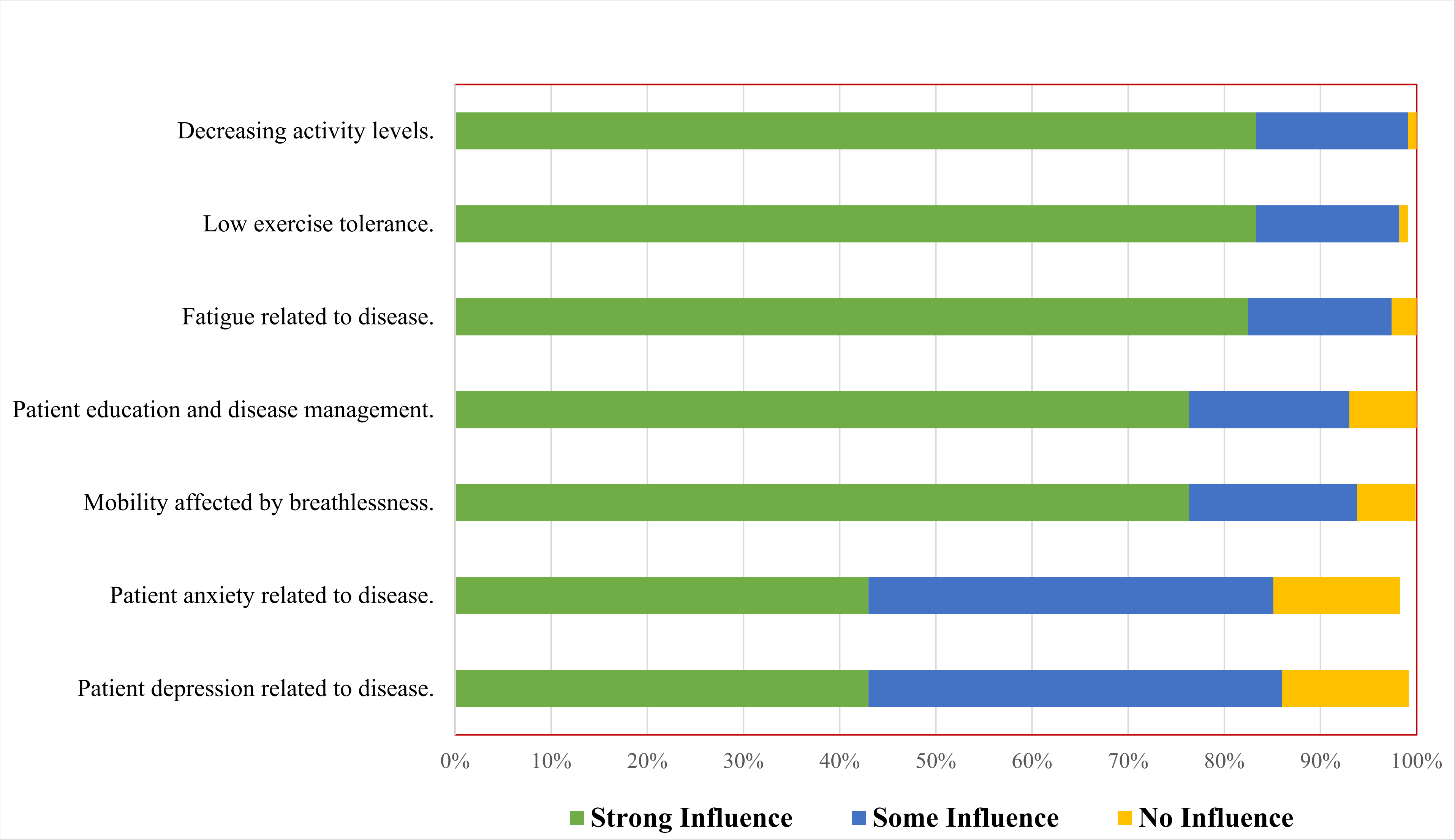

Figure S2: Barriers of not referring patients with Heart Failure to cardiopulmonary rehabilitation from cardiac doctors' perspective, using influence graded as no, some or strong influence. (n=114)

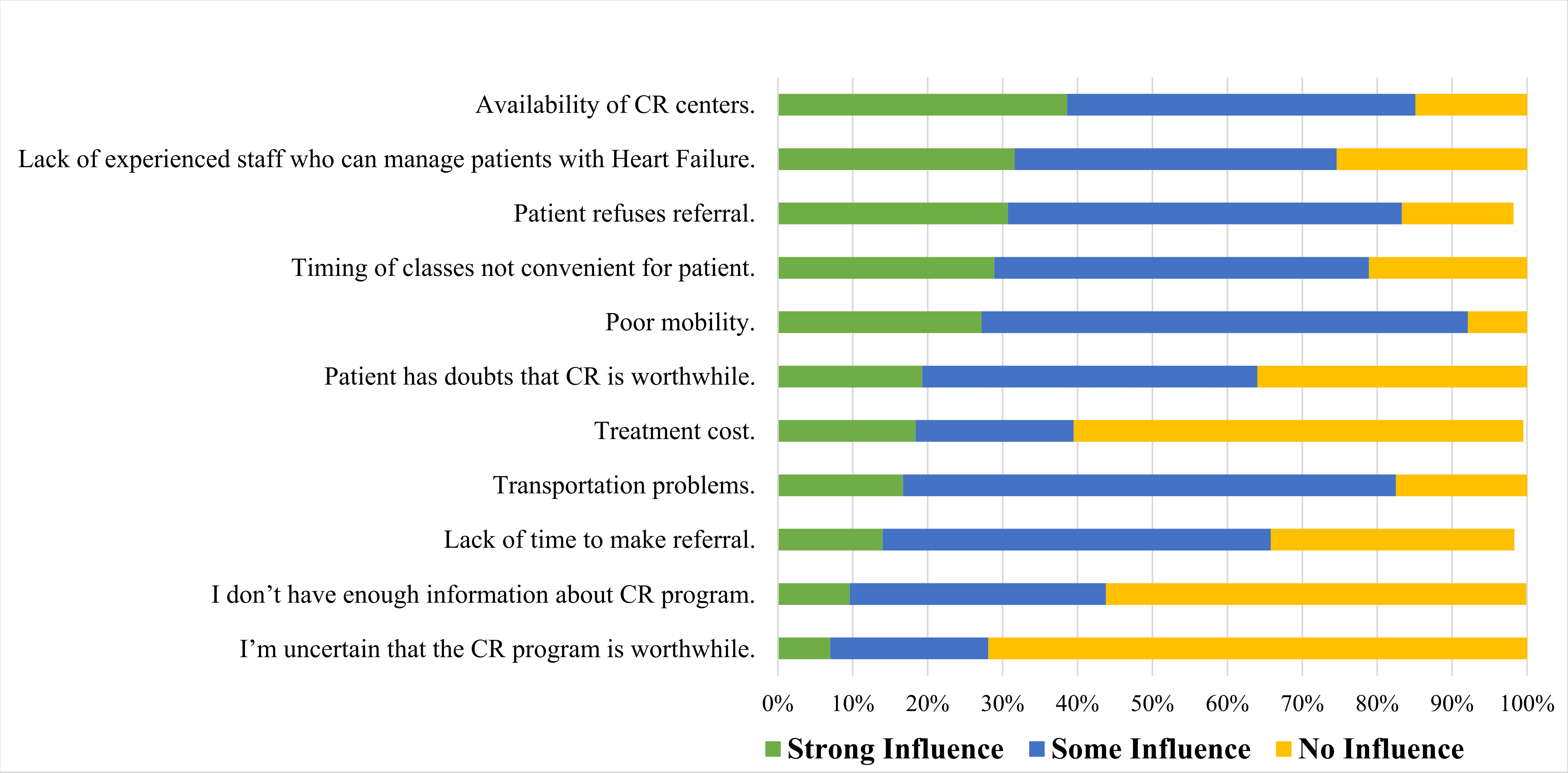

Figure S3: Patient-related factors that influence referral decision to cardiopulmonary rehabilitation from general physicians' perspective, using strong, some or no influence as a grading tool (n=399).

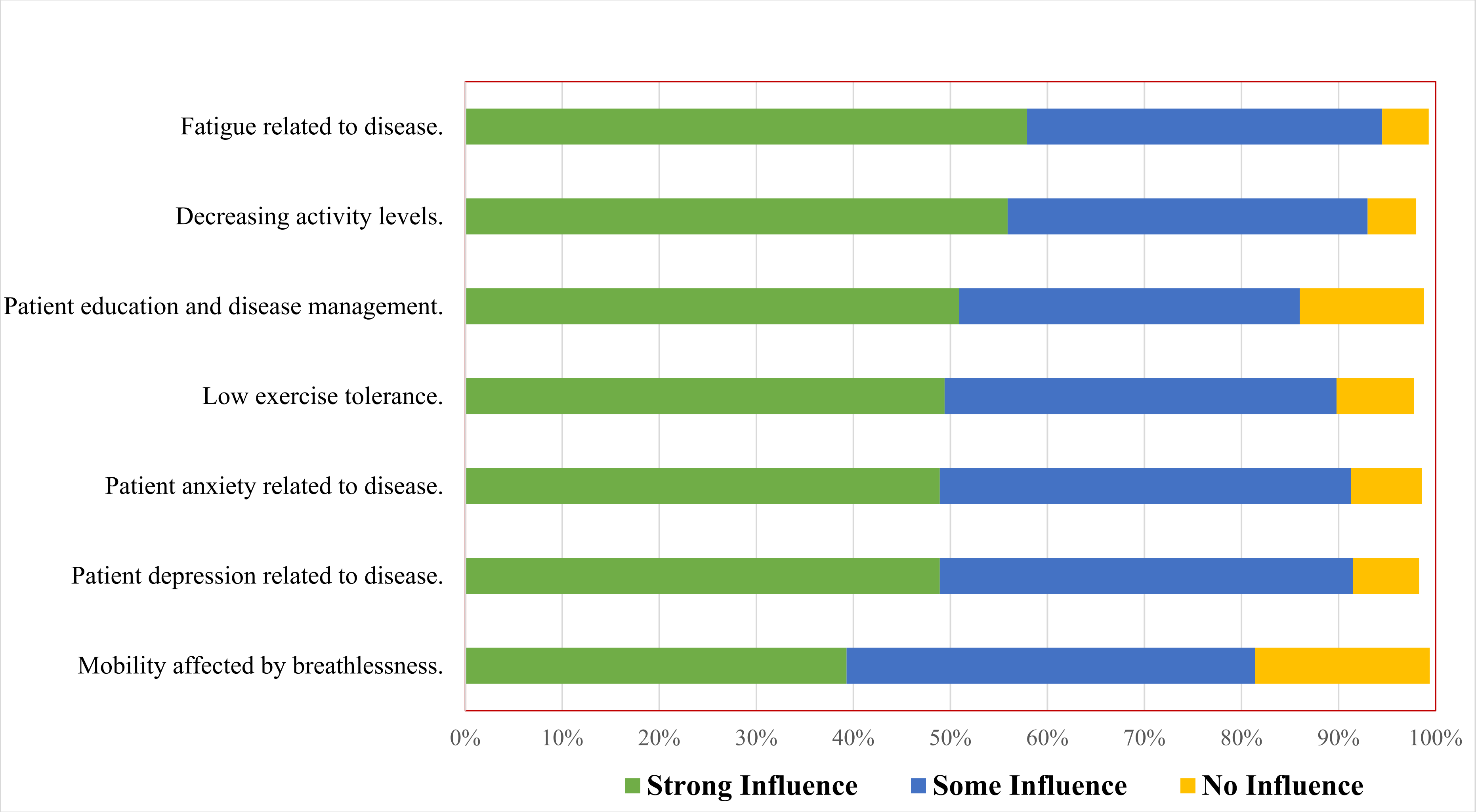

Figure S4: Barriers of not referring patients with Heart Failure to cardiopulmonary rehabilitation from general physicians' perspective, using influence graded as no, some or strong influence. (n=399)

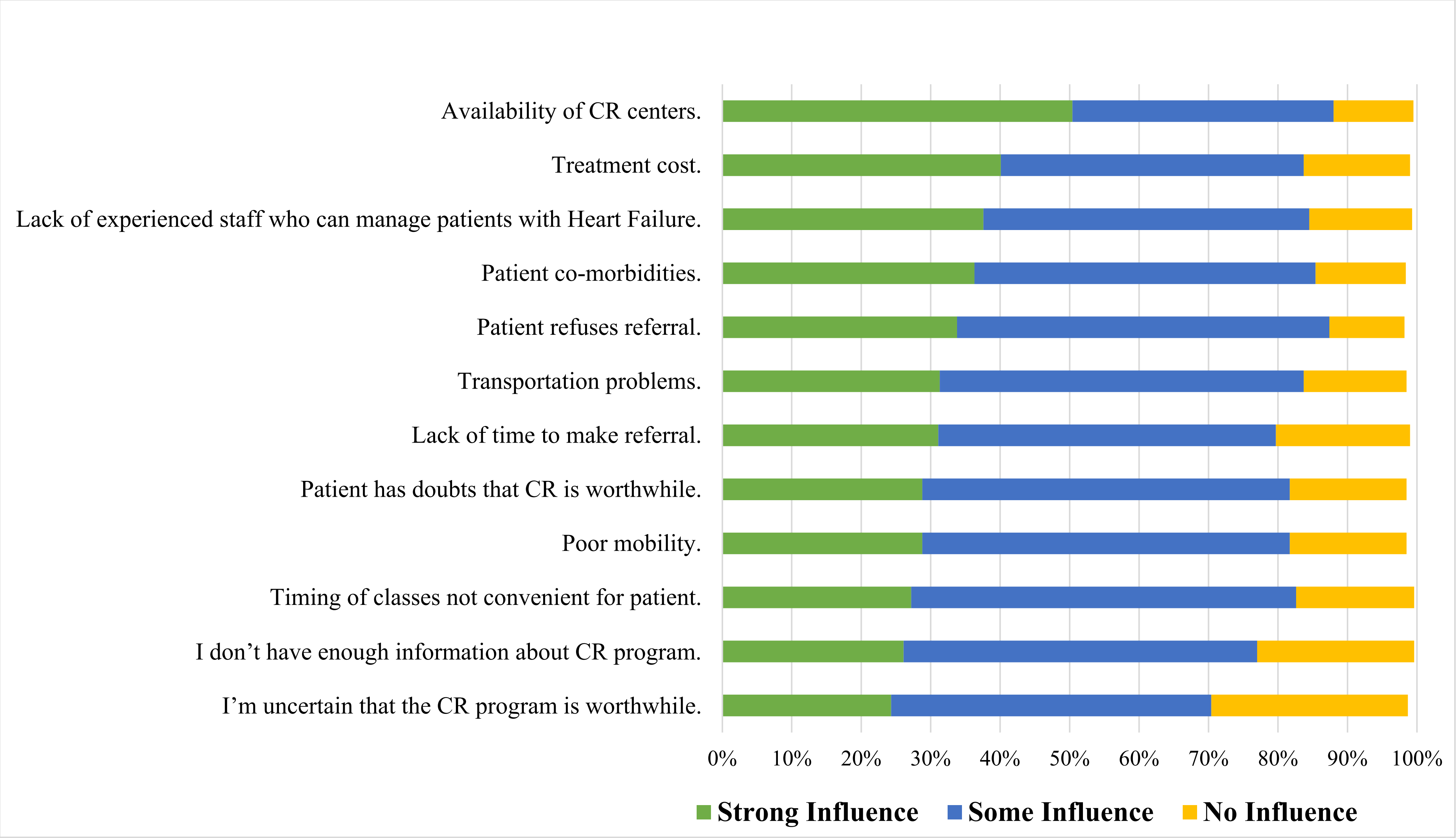

Supplement: Supplementary file 1 [file ijerph-19-15208-s001.zip › ijerph-1940811-supplementary.pdf]
